# Selective Degradation of Host RNA Polymerase II Transcripts by Influenza A Virus PA-X Host Shutoff Protein

**DOI:** 10.1371/journal.ppat.1005427

**Published:** 2016-02-05

**Authors:** Denys A. Khaperskyy, Summer Schmaling, Jonah Larkins-Ford, Craig McCormick, Marta M. Gaglia

**Affiliations:** 1 Department of Microbiology and Immunology, Dalhousie University, Halifax, Nova Scotia, Canada; 2 Department of Molecular Biology and Microbiology and Graduate Program in Molecular Microbiology, Tufts University School of Medicine, Boston, Massachusetts, United States of America; Icahn School of Medicine at Mount Sinai, UNITED STATES

## Abstract

Influenza A viruses (IAVs) inhibit host gene expression by a process known as host shutoff. Host shutoff limits host innate immune responses and may also redirect the translation apparatus to the production of viral proteins. Multiple IAV proteins regulate host shutoff, including PA-X, a ribonuclease that remains incompletely characterized. We report that PA-X selectively targets host RNA polymerase II (Pol II) transcribed mRNAs, while sparing products of Pol I and Pol III. Interestingly, we show that PA-X can also target Pol II-transcribed RNAs in the nucleus, including non-coding RNAs that are not destined to be translated, and reporter transcripts with RNA hairpin structures that block ribosome loading. Transcript degradation likely occurs in the nucleus, as PA-X is enriched in the nucleus and its nuclear localization correlates with reduction in target RNA levels. Complete degradation of host mRNAs following PA-X-mediated endonucleolytic cleavage is dependent on the host 5’->3’-exonuclease Xrn1. IAV mRNAs are structurally similar to host mRNAs, but are synthesized and modified at the 3’ end by the action of the viral RNA-dependent RNA polymerase complex. Infection of cells with wild-type IAV or a recombinant PA-X-deficient virus revealed that IAV mRNAs resist PA-X-mediated degradation during infection. At the same time, loss of PA-X resulted in changes in the synthesis of select viral mRNAs and a decrease in viral protein accumulation. Collectively, these results significantly advance our understanding of IAV host shutoff, and suggest that the PA-X causes selective degradation of host mRNAs by discriminating some aspect of Pol II-dependent RNA biogenesis in the nucleus.

## Author Summary

All viruses depend on host components to convert viral mRNAs into proteins. Several viruses, including influenza A virus, encode factors that trigger RNA destruction. The influenza A virus factor that serves in this capacity is known as PA-X. PA-X limits accumulation of host mRNAs and proteins in infected cells and suppresses host responses to infection, but to date its precise mechanism of action remains obscure. Here we report that PA-X selectively targets cellular mRNAs, while sparing viral mRNAs, thereby compromising host gene expression and ensuring priority access of viral mRNAs to the protein synthesis machinery. We demonstrate that complete degradation of mRNAs cut by PA-X is dependent on the host factor Xrn1 and that PA-X likely works in the cell’s nuclei. Interestingly, PA-X targeting appears to be selective for products of host RNA polymerase II, and canonical mRNA processing is required for cleavage. Even though viral mRNAs are spared from PA-X-mediated degradation, PA-X-deficient viruses displayed defects in the synthesis of certain viral mRNAs and decreased viral protein accumulation. Thus, PA-X-mediated host shutoff influences the efficiency of viral gene expression. These studies significantly advance our understanding of this important viral host shutoff protein and may provide future opportunities to limit the pathogenesis of influenza A virus infection.

## Introduction

Inhibition of host gene expression, termed “host shutoff”, is thought to enable viruses to simultaneously inhibit innate immune responses and provide preferential access for viral mRNAs to the cellular translation machinery. Influenza A virus (IAV) has long been known to carry out host shutoff, and multiple shutoff mechanisms have been reported for this virus, including translation blockade [1], inhibition of polyadenylation and nuclear export of host pre-mRNAs by the IAV NS1 protein [2], and degradation of the host RNA polymerase II complex [3]. Because some of these mechanisms are specific to certain IAV strains [4], it has long been suspected that more universal IAV host shutoff mechanisms exist. The recent discovery of the highly conserved RNA endonuclease PA-X [5] has prompted re-examination of established models of IAV host shutoff. Viruses from several divergent families use virus-encoded RNA endonucleases to broadly degrade host mRNAs and reduce host protein production [5–9]. Although host shutoff ribonucleases (RNases) generally have broad specificity *in vitro*, several studies have shown unexpected selectivity for different types of host transcripts [10–15]. PA-X limits accumulation of host mRNAs and proteins in infected cells and suppresses host responses to infection [5,16–19], but the mechanistic determinants of selectivity, cleavage and degradation are not yet known.

IAV is a negative strand RNA virus with a genome consisting of eight segments. PA-X protein is encoded on IAV genome segment 3, which also produces polymerase acidic protein (PA), one of the three subunits of the viral RNA-dependent RNA polymerase (RdRp). It is generated by ribosomal pausing on a rare CGU codon that results in a +1 frameshift and read-through of an alternative open reading frame (ORF) [5,20]. PA-X comprises the amino-terminal 191 amino acids of the polymerase subunit PA fused to a carboxy-terminal domain (termed “X-ORF”) of either 41 or 61 amino acids that result from the frameshift [5,21]. Consequently, PA-X lacks the carboxy-terminal domain of PA responsible for its recruitment into the RdRp complex. The shared PA/PA-X amino-terminal domain includes an RNA endonuclease domain that is required for PA-X shutoff activity [5]. Thus, PA-X has a function analogous to host-shutoff proteins from other viruses that trigger RNA degradation. These factors include SARS coronavirus nsp1 [9,22], herpes simplex virus 1 (HSV-1) vhs [6,23] and Kaposi’s sarcoma-associated herpesvirus (KSHV) SOX [8]. In general, these proteins are unrelated at a molecular level, although both PA-X and the SOX family of proteins belong to the PD-(D/E)XK nuclease superfamily [24–28]. All known host-shutoff RNases use a similar mechanism of action, causing endonucleolytic cleavage of the RNA and relying on host enzymes to complete RNA degradation [14]. Moreover, they are all selective for translatable RNA polymerase II (Pol II) transcripts, but spare non-coding RNAs (ncRNAs) synthesized by Pol I and Pol III [14]. This selectivity has been linked to the process of translation or loading into translation initiation complexes [14,22,29–31].

RdRp-transcribed IAV mRNAs share essential features with host mRNAs like a 5’ 7-methyl guanosine (m^7^G) cap and a 3’ poly-adenylate (poly(A)) tail. 5’ m^7^G caps are acquired by “cap-snatching”, whereby the PA subunit cleaves nascent host Pol II-transcribed RNAs at a position 10–14 nucleotides downstream from the 5’ cap [32]. The RdRp complex uses these fragments to prime viral mRNA synthesis. The poly(A) tail is generated by RdRp “stuttering”, allowing reiterative copying of a short poly-uridine sequence at the 5’ end of the template genome segment [33]. The acquisition of m^7^G caps and poly(A) tails allows efficient loading of ribosomes and translation of IAV mRNAs; however, the similarity between host and viral mRNAs raises the question of their susceptibility to PA-X-mediated degradation.

Here, we demonstrate that PA-X selectively targets host RNA transcribed by the RNA Pol II complex for cleavage, and degradation is completed by the host 5’->3’ exonuclease Xrn1. By contrast, host transcripts generated by other Pol complexes resist PA-X-mediated degradation. Interestingly, selective targeting is not linked to translation, as we observe that PA-X also degrades non-coding Pol II transcripts, and may instead be linked to distinct features of Pol II transcript biogenesis in the nucleus. Consistent with these observations, we show that PA-X is recruited to the nucleus via X-ORF interactions that involve highly conserved basic residues previously shown to be important for the shutoff function [34]. Accordingly, we find that IAV mRNAs generated by viral polymerase complexes are intrinsically resistant to PA-X-mediated degradation. In addition, we demonstrate that PA-X is required for efficient translation of viral mRNAs, as viral protein accumulation is significantly diminished in cells infected with PA-X deficient viruses. Taken together these findings suggest that IAV PA-X hijacks cellular RNA biogenesis processes to direct the degradation of host RNAs, and that the distinct biogenesis mechanism for viral mRNAs provides a convenient way to discriminate host and viral products. Thus, although PA-X shares some mechanistic properties with other host shutoff RNases, it also displays distinctive features that advance our understanding of host shutoff.

## Results

### PA-X selectively degrades Pol II-transcribed RNAs

**Fig 1:**
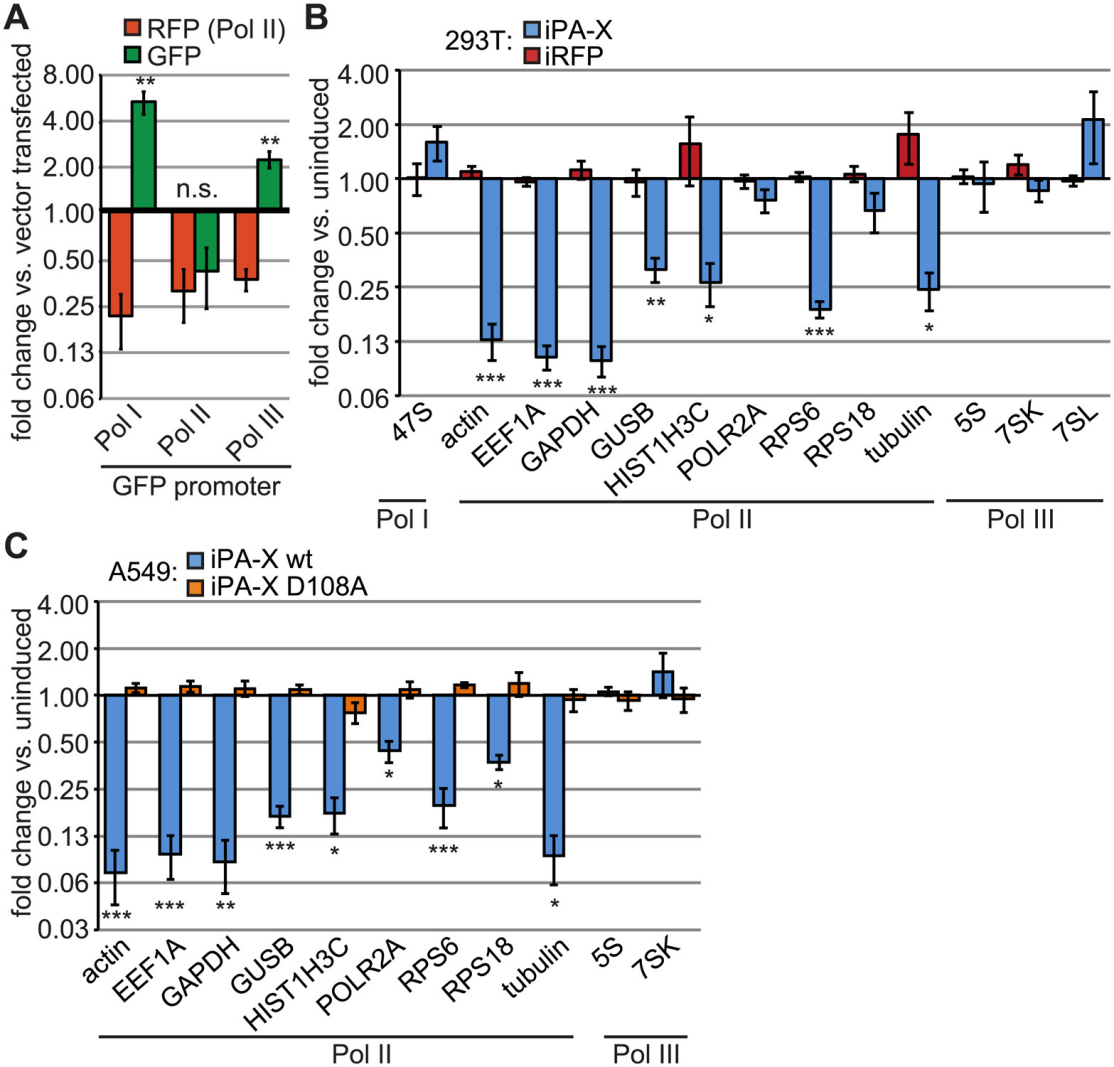
RNA targeting by PA-X is dependent on the host RNA polymerase complex that transcribes the RNA. (A) HEK 293T cells were transfected with a Pol II-driven RFP reporter, the indicated GFP reporters expressed from Pol I, Pol II or Pol III-driven promoters, and either PA-X-myc or an empty vector. Total cellular RNA was extracted and RFP, GFP and 18S RNA levels were analyzed by RT-qPCR. Reporter expression normalized against 18S RNA levels is plotted as fold change between PA-X-expressing vs. vector transfected cells. n.s.,**,*** = *p* value (Student’s *t*-test) > 0.05 or < 0.01, 0.001 for fold change of GFP vs. fold change of RFP. (B-C) HEK 293T (B) or A549 (C) cells expressing doxycycline-inducible wild-type PA-X-myc (“iPA-X wt”), a catalytically inactive mutant (“iPA-X D108A”) or RFP (“iRFP”) were treated with doxycycline for 18 h to induce expression of PA-X or RFP. Total cellular RNA was extracted and RT-qPCR was performed to measure levels of the indicated endogenous RNAs. Endogenous RNA levels were normalized by 18S rRNA levels and are plotted as fold change in induced (PA-X- or RFP-expressing) vs. uninduced cells. HEK293T iPA-X line #7 and A549 iPA-X wt line #10 and D108A line #8 were used for this figure. Data on additional clonal lines is presented in S1 Fig. *,**,*** = *p* value (Student’s *t*-test) < 0.05, 0.01, 0.001 vs. 293T iRFP (B, n = 5) or A549 iPA-X D108A line #8 (C, n = 3). Error bars = s.e.m.

Diverse RNA species are generated by three host DNA-dependent RNA polymerases; Pol II transcribes mRNAs and some non-coding RNAs (ncRNAs), whereas Pol I transcribes the rRNA precursor 47S and Pol III transcribes a variety of short ncRNAs including the 5S rRNA. To determine whether PA-X could trigger the degradation of distinct host RNA species, we transfected HEK 293T cells with plasmid reporters encoding an RFP gene driven by a Pol II promoter and a GFP gene under the control of a Pol I-, Pol II-, or Pol III-driven promoter. We observed that ectopic expression of PA-X derived from the A/PuertoRico/8/34 H1N1 (PR8) virus together with these reporters consistently inhibited the accumulation of Pol II-driven RFP and GFP transcripts, whereas Pol I and Pol III-driven GFP transcripts accumulated to high levels (Fig 1A). These data suggest that PA-X activity is specific for Pol II transcripts.

To determine whether PA-X could similarly selectively target endogenous Pol II-driven transcripts, we constructed HEK 293T- and A549-based cell lines that stably express PA-X in a doxycycline-inducible manner (iPA-X cells). In comparison to an inducible RFP control, PA-X expression in the HEK 293T cell lines dramatically reduced endogenous mRNA levels for actin, EEF1A, GAPDH, GUSB, HIST1H3C, tubulin and RPS6, but had a much smaller effect on RPS18 and POLR2A mRNAs (Fig 1B). By contrast, consistent with our results with reporter constructs (Fig 1A), PA-X did not decrease the levels of the Pol I transcript 47S or several Pol III transcripts (5S, 7SK and 7SL). Both the specificity for Pol II transcripts and the variable level of down-regulation of different mRNAs were also observed in the A549 lung carcinoma cell line (Fig 1C). In addition, expression of the PA-X RNase domain catalytic mutant D108A [5,35] in A549 cells did not affect RNA levels (Fig 1C). This result indicates that the mRNA down-regulation is likely due to increased degradation, as it requires an intact RNase domain. Multiple independently isolated HEK 293T and A549 cell lines generated similar results (S1A and S1B Fig). Thus, PA-X expressed in isolation decreases the levels of a broad range of host mRNAs with varying efficiency, but Pol I- and Pol III-transcribed RNAs resist degradation, pointing at a mechanism of action similar to that of previously described host-shutoff RNases from other viruses [14].

### PA-X-mediated RNA degradation requires the host RNase Xrn1

**Fig 2:**
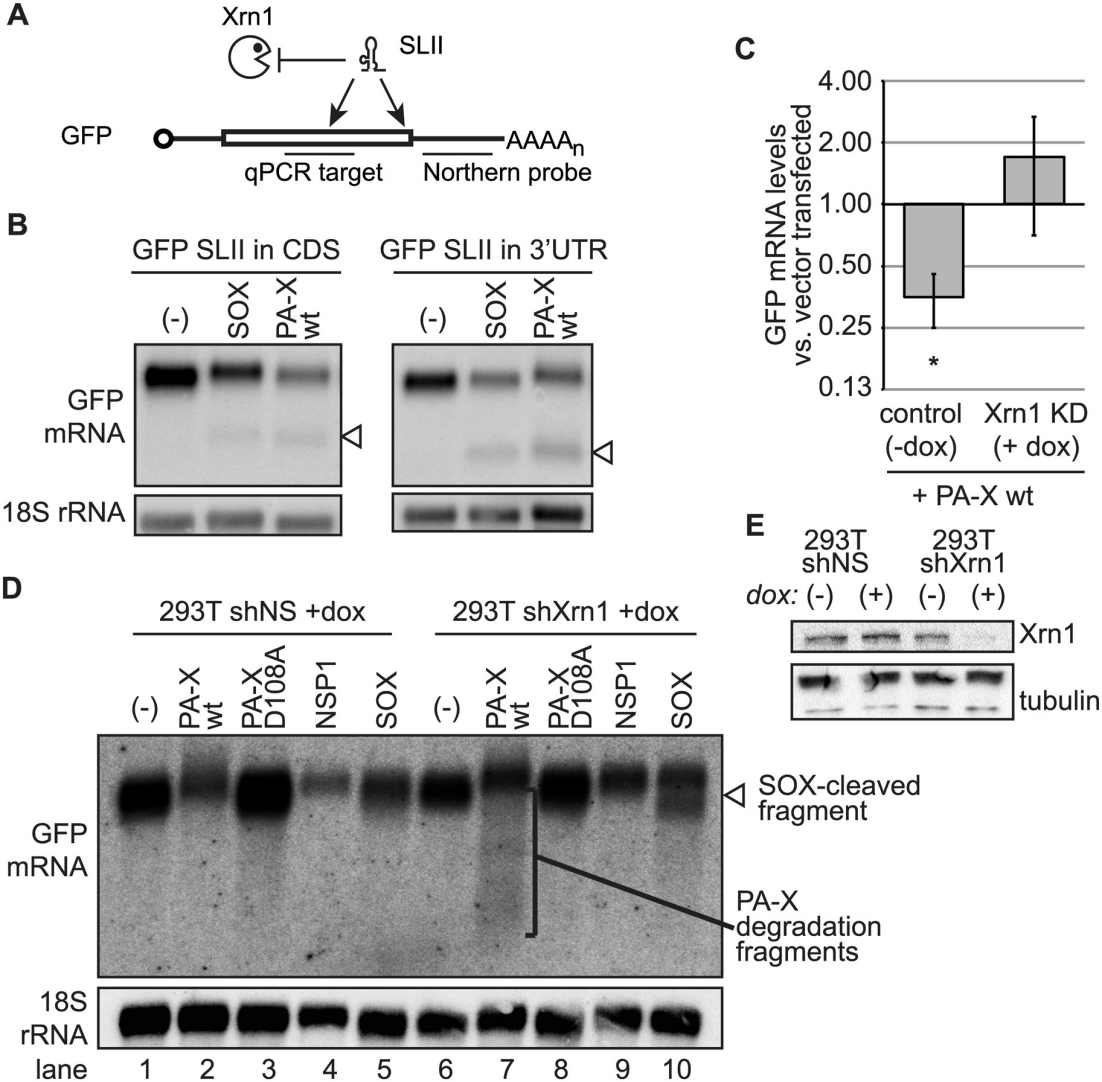
Complete degradation of mRNA by PA-X is dependent on the cellular exonuclease Xrn1. (A) Diagram showing GFP constructs with a flaviviral Xrn1-blocking element (SLII) introduced at different positions. (B) HEK 293T cells were co-transfected with GFP-SLII constructs and PA-X, SOX, or empty vector (“-”). Total cellular RNA was extracted and northern blotted using probes against the 3’UTR of the GFP reporter or against 18S (as a loading control). Arrowheads indicate RNA fragments protected from degradation by the SLII sequence. (C-E) HEK 293T shXrn1 or shNS cells were treated with doxycycline (+dox) for four days to induce shRNAs against Xrn1 or non-specific control shRNAs (shNS), or were left untreated (-dox). Three days after shRNA induction, cells were transfected with a GFP reporter plasmid and the indicated construct or empty vector (“-”). Total cellular RNA was extracted and subjected to northern blot analysis or RT-qPCR. (C) GFP mRNA levels were analyzed by RT-qPCR and normalized against levels of 18S rRNA (also detected by RT-qPCR). Normalized GFP expression is plotted as fold change in PA-X-expressing vs. vector-transfected cells. * = *p* value (one-sample *t*-test) < 0.05. (D) Northern blot probes directed against the 3’UTR of the GFP reporter or 18S (as a loading control) were used to detect RNA levels. The bracket denotes PA-X degradation fragments and the arrowhead indicates the RNA fragment generated by SOX cleavage. A representative image is shown. (E) Levels of Xrn1 for experiment in panel D were assessed by western blot, using tubulin as a loading control. This western blot is also representative of the Xrn1 knock-down observed in samples used for panel C and other repeats of the northern blot.

We previously found that herpesvirus and coronavirus host shutoff endonucleases employ analogous mechanisms to trigger widespread RNA degradation, whereby endonuclease cleavage of target RNA is followed by processive 5’->3’ degradation by the host exonuclease Xrn1 [14]. To determine whether PA-X requires host 5’->3’ exonucleases to complete RNA degradation, we tested plasmid reporters that contain a pseudo-knot forming sequence from West Nile virus (SLII [36]) in either the GFP coding region or the 3’ UTR (Fig 2A); the presence of the SLII element leads to protection of the downstream RNA from digestion by 5’->3’ exonucleases [14,29]. Northern blotting using a probe against the 3’ untranslated region (UTR) of GFP showed that ectopic expression of PA-X reduced the levels of full-length GFP mRNA and that a SLII-protected species appeared, which indicated that host 5’->3’ exonucleases were required for full degradation of target mRNAs (Fig 2B). To determine whether Xrn1 in particular is required for full PA-X-initiated RNA degradation, Xrn1 expression was silenced in HEK 293T cells by doxycycline-inducible shRNA expression (Fig 2E). As expected from previous observations (Fig 1A), ectopic PA-X expression reduced GFP mRNA levels in control cells, but induction of Xrn1 shRNA expression reversed this phenotype (Fig 2C). Using a northern blotting approach with a probe specific for GFP mRNA, we found that Xrn1 knock-down in wild-type PA-X-expressing cells caused the appearance of a heterogeneous population of partially digested GFP mRNA products (Fig 2D, lane 7). This is consistent with a model in which PA-X cleavage occurs throughout the length of the RNA with no discrete target site, followed by full digestion by host 5’-3’ exonucleases. As expected, neither a decrease in full-length GFP RNA levels nor the GFP fragments were detected when the D108A catalytic mutant of PA-X was expressed (Fig 2D, lanes 3 and 8). The lack of an apparent discrete primary endonuclease cleavage site makes PA-X unique amongst host-shutoff endonucleases studied to date, all of which either reveal sequence-specific cleavage sites on mRNAs, or target the 5’ end of transcripts [14,15,22,29,37]. Indeed, analysis of KSHV SOX cleavage products in Xrn1-deficient cells reveals the accumulation of a specific degradation product, reflecting cleavage at a discrete location in the mRNA (Fig 2D, lane 10), consistent with published reports [29]. Moreover, SARS nsp1, which is reported to cut RNAs close to the 5’ end [22] did not cause the appearance of partially digested GFP mRNA products (Fig 2D, lane 9). Together, these data indicate that PA-X degrades host mRNAs in concert with Xrn1 and perhaps other cellular exonucleases, and unlike other host-shutoff endonucleases, lacks obvious sequence or location specificity, consistent with existing *in vitro* data [38].

### Translation is not required for PA-X-mediated degradation of Pol II transcripts

**Fig 3:**
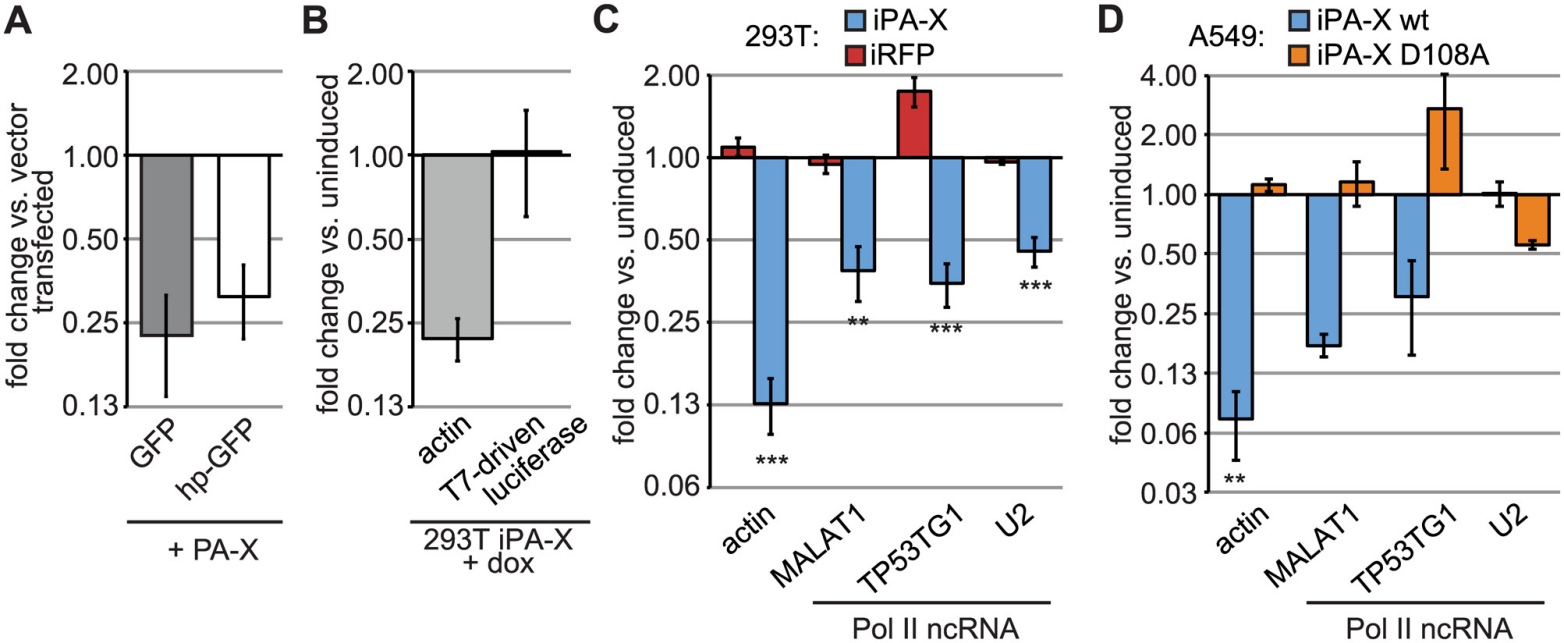
PA-X targets Pol II-transcribed mRNAs that are not translated. (A) HEK 293T cells were transfected with Pol II-driven GFP reporters and either PA-X or an empty vector. The hp-GFP construct contains a hairpin close to the 5’ end of the GFP mRNA that blocks its translation. Total cellular RNA was extracted and GFP mRNA levels were measured by RT-qPCR. GFP expression normalized by 18S rRNA abundance is plotted as fold change in PA-X vs. vector-transfected cells. Error bars = st. dev. (B) 293T iPA-X cells were transfected with a T7 polymerase expressing construct and an EMCV IRES-containing luciferase reporter driven by the T7 promoter, which results in T7-mediated luciferase RNA transcription. PA-X expression was induced 5 h after transfection by doxycycline addition for 18 h. Total cellular RNA was extracted and the levels of the luciferase reporter and endogenous actin mRNA were measured by RT-qPCR. RNA levels were normalized by 18S rRNA abundance and are plotted as fold change in induced (PA-X-expressing) vs. uninduced cells. (C-D) HEK 293T (C) or A549 (D) cells expressing doxycycline-inducible wild-type PA-X-myc (“iPA-X wt”), a catalytically inactive mutant (“iPA-X D108A”) or RFP (“iRFP”) were treated with doxycycline for 18 h to induce expression of PA-X. Total cellular RNA was extracted and the levels of the indicated endogenous RNAs were measured by RT-qPCR. Levels of actin mRNAs (also shown in Fig 1) and Pol II-driven non-coding RNAs were normalized to 18S rRNA levels and are plotted as fold change in induced (PA-X- or RFP-expressing) vs. uninduced cells. HEK293T iPA-X line #7 and A549 iPA-X wt line #10 and D108A line #8 were used for this figure. Data on additional clonal lines is presented in S2B and S2C Fig *,**,*** = *p* value (Student’s *t*-test) < 0.05, 0.01, 0.001 vs. 293T iRFP (C, n = 5) or A549 iPA-X D108A #8 (D, n = 3). Error bars = s.e.m.

Many Pol II-transcribed RNAs are translated. Association with components of the translation apparatus has been shown or proposed to be a determinant of selective mRNA targeting by other host-shutoff RNases, such as HSV-1 vhs [30,31] and KSHV SOX [29]. Moreover, SARS nsp1 requires active translation of RNAs for degradation [22]. To investigate the relationship between translation and RNA targeting by PA-X, we employed a Pol II-driven reporter that is not translated (S2A Fig) due to the insertion of a hairpin close the 5’ cap (hp-GFP). This reporter mRNA associates with the translation initiation machinery, but cannot be translated because the hairpin blocks ribosome association and/or scanning [39]. We observed that PA-X decreased the levels of the hp-GFP and a control GFP mRNAs to a similar extent, suggesting that targeting is independent of mRNA translation (Fig 3A). Conversely we used a dual-construct T7 polymerase system to direct transcription of luciferase RNA by overexpressed T7 polymerase [40], rather than cellular Pol II. The T7-synthesized luciferase RNA is actively translated due to the presence of an EMCV internal ribosome entry site (S2B Fig), but luciferase mRNA levels are unaffected by PA-X (Fig 3B). These results indicate that synthesis by RNA Pol II, rather than translatability of the RNA, is a major determinant for targeting by PA-X.

In addition to mRNAs, Pol II also transcribes several ncRNAs, including the long ncRNAs MALAT1 and TP53TG1 and the precursor of the small nuclear RNA U2. Based on our reporter data (Fig 3A and 3B), we hypothesized that Pol II transcribed ncRNAs may also be targeted by PA-X. Consistent with this hypothesis, we found that levels of the MALAT1 and TP53TG1 ncRNAs were reduced in 293T (Fig 3C, S2C Fig) and A549 iPA-X cell lines (Fig 3D, S2D Fig). The effect of PA-X on the levels of U2 was variable; however this small nuclear RNA was unaffected in A549 iPA-X cells and some 293T iPA-X clones (Fig 3C and 3D, S2C and S2D Fig). Half-life measurements for the actin mRNA and MALAT1 ncRNA in the presence of wild-type and catalytically inactive PA-X confirmed that PA-X directly affects the stability of the different RNA species (S2E Fig). These results indicated that PA-X targets at least some Pol II synthesized ncRNAs for degradation and that, unlike herpesviral host shutoff endonucleases and SARS nsp1, association with the protein synthesis machinery is not a prerequisite for targeting by PA-X.

### Pol II-transcribed host RNAs are selectively degraded by PA-X during IAV infection

**Fig 4:**
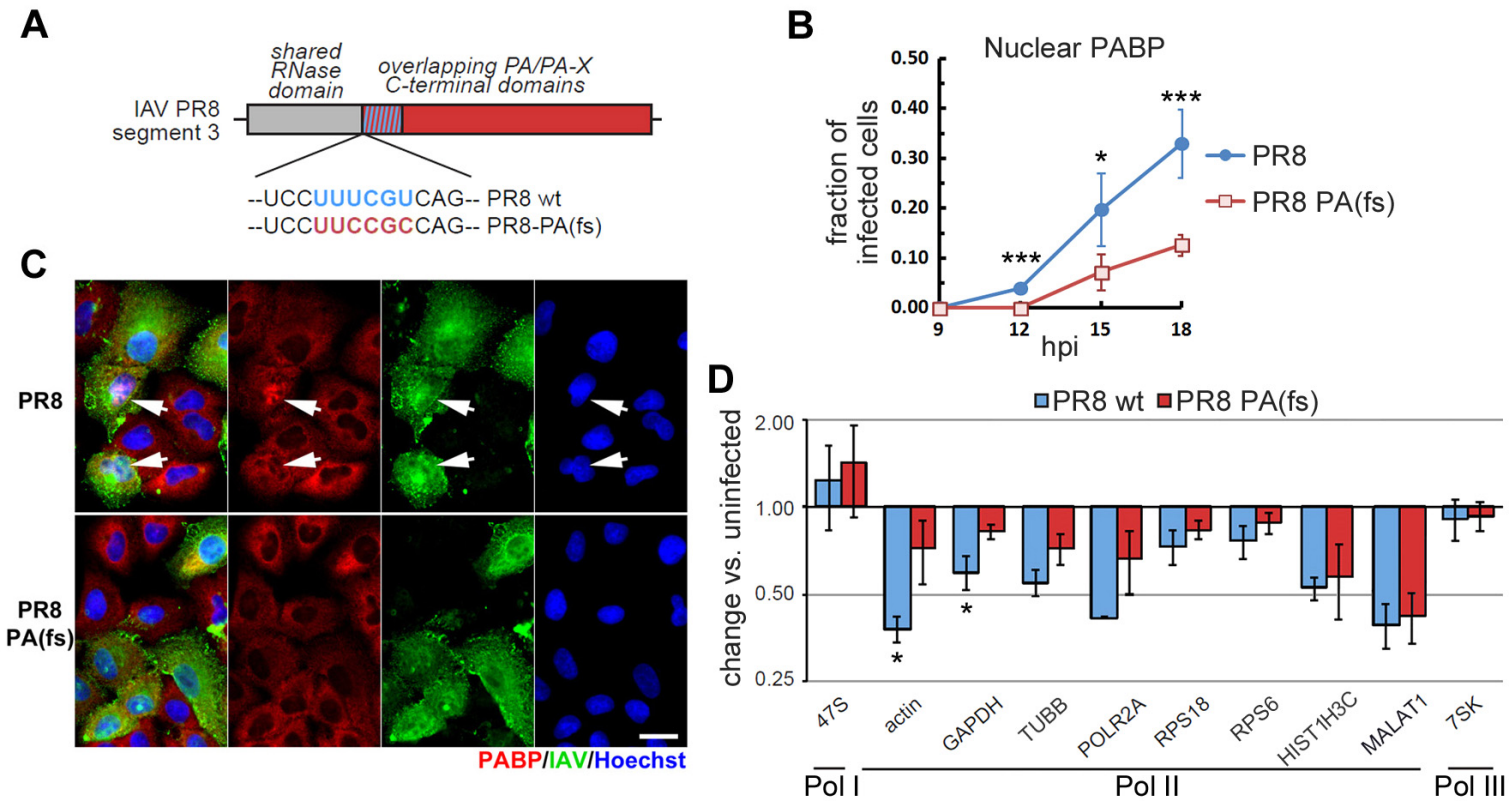
PA-X selectively degrades Pol II transcripts in infected cells. (A) Schematic representation of the PA gene of IAV showing the sequence of the frameshift site in the wild-type PR8 and the substitutions made to generate PR8-PA(fs) mutant virus. (B-D) A549 cells were infected with PR8 or PR8-PA(fs) at a multiplicity of infection (MOI) of 1. The fraction of infected cells displaying nuclear PABP was quantified (B) by immunofluorescence staining (C) of cells fixed at the indicated time points. A minimum of 200 cells were quantified in each sample. (D) Total RNA was harvested at 12 hours post-infection (hpi) and RT-qPCR was performed to measure levels of the indicated endogenous RNAs. Endogenous RNA levels were normalized to 18S rRNA levels and plotted as fold change in infected vs. uninfected cells. Error bars = st. dev. from three independent biological replicates. *,*** = *p* value < 0.05, 0.005 for PR8-PA(fs)- vs. PR8-infected cells.

To determine whether PA-X selectively targets Pol II-transcribed RNA in the context of IAV infection, A549 cells were infected with PR8 or a mutant PR8-PA(fs) virus that should not produce PA-X due to codon optimization that prevents ribosome pausing and frameshifting (Fig 4A). As we previously reported [41], at later times post-infection PR8 virus causes dramatic depletion of cytoplasmic polyadenylated RNA which drives the nuclear relocalization of poly(A) binding protein (PABP). By contrast, in cells infected with the PR8-PA(fs) mutant virus, nuclear PABP relocalization is significantly delayed, and only becomes detectable at 12 hours post-infection (hpi, Fig 4B and 4C). The fact that PABP relocalization, a known PA-X-dependent phenotype, is markedly reduced in cells infected with the PR8-PA(fs) virus confirms that this virus is PA-X deficient; however, we note that nuclear PABP relocalization was still observed at later times post-infection. Other host shutoff mechanisms and/or leaky expression of PA-X in PR8-PA(fs) virus-infected cells may cause late nuclear localization of PABP. For this reason, we selected the 12 hpi time point for the analyses of host transcript levels. Consistent with our observations from PA-X ectopic expression experiments (Fig 1, S1 Fig), actin and GAPDH mRNA levels were reduced in a PA-X-dependent manner (Fig 4D) in PR8 IAV infected cells. We also detected a decrease in the levels of tubulin, POLR2A, and, to a lesser extent, RPS6 and RPS18, but in these cases the decrease was only partially dependent on PA-X function, and may be due in part to other host-shutoff mechanisms. Interestingly, the Pol II-transcribed ncRNA MALAT1 and the histone mRNA HIST1H3C were strongly down-regulated in both PR8 wt and PR8-PA(fs) infected cells (Fig 4D), suggesting that these RNAs are subject to regulation by other viral proteins. In contrast to our results with Pol II transcripts, the levels of Pol I and Pol III-transcribed ncRNAs (47S and 7SK, respectively) were not altered in PR8 or PR8-PA(fs) infected cells. Collectively, these data show that during an IAV infection, PA-X selectively targets Pol II-transcribed RNAs for degradation.

### PA-X is required for efficient IAV protein accumulation and viral replication

**Fig 5:**
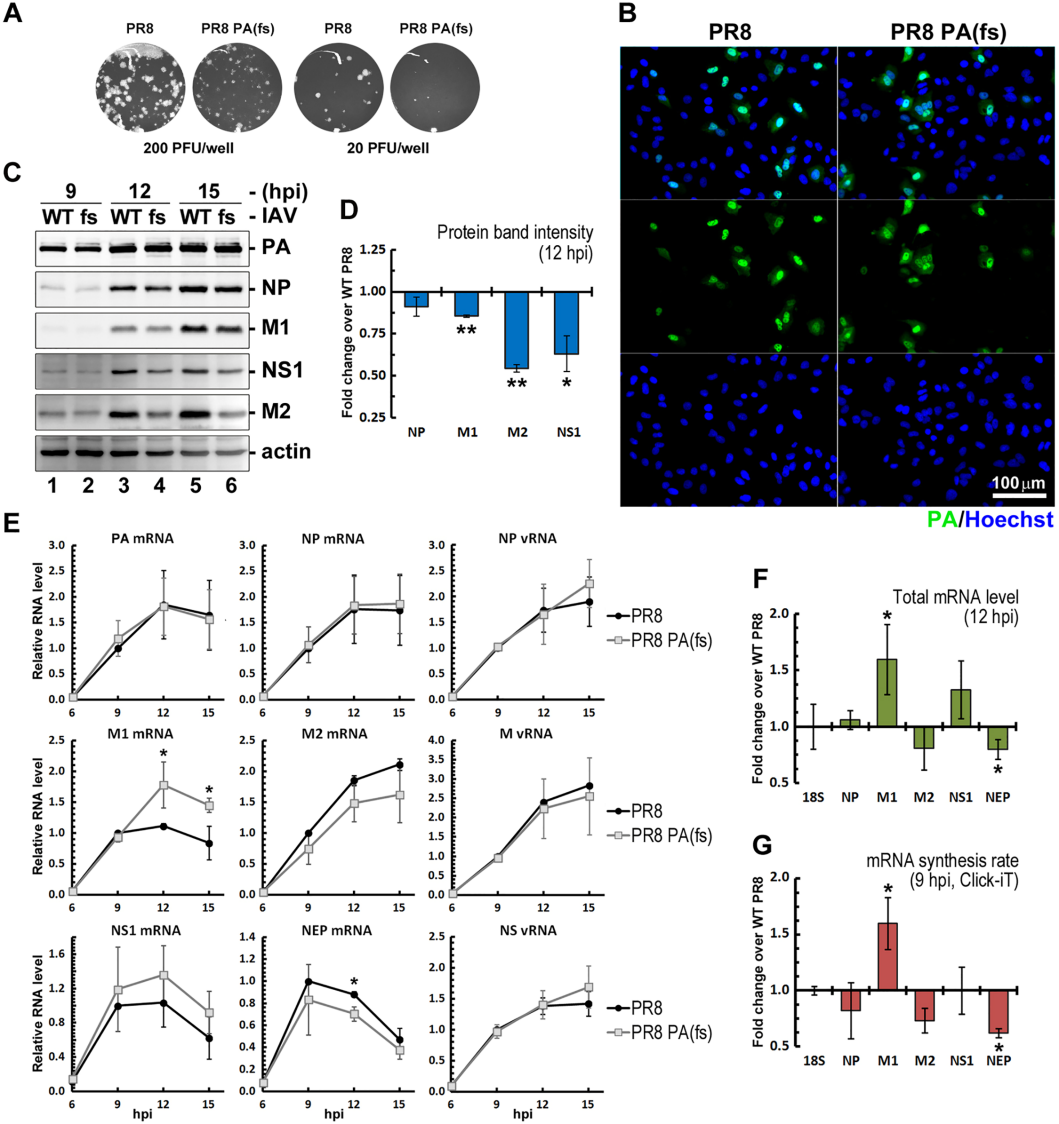
PA-X activity facilitates viral protein synthesis. (A) Crystal violet staining of plaques generated by wild-type PR8 and the PR8-PA(fs) mutant viruses in Vero cells, showing a decrease in plaque size for PA-X-deficient mutant compared to wild-type virus plaques. (B-D) A549 cells were infected with PR8 or PR8-PA(fs) IAV at a MOI of 0.25. (B) At 12 hpi cells were fixed and the infected cells were identified by immunofluorescence staining for IAV PA protein (green) to confirm that infection rates were highly similar between the two virus inoculums. Cell nuclei were stained with Hoechst dye (blue). (C) Viral protein accumulation was determined by western blotting of the whole cell lysates collected at the indicated time points. (D) Band intensities from (C) were quantified using ImageJ software (NIH) from two independent experiments. Error bars = st. dev. (E) Total RNA was isolated from cells infected as in (A-C) and the mRNA and vRNA levels for a subset of IAV genes were measured by RT-qPCR. Data was normalized to 18S rRNA and values plotted relative to 9 hpi levels in wild-type virus infected cells. (F) The levels of the indicated viral mRNAs at 12 hpi from (E) were plotted as fold change in PR8-PA(fs) infected cells vs. PR8 wild-type infected cells. (G) Rates of RNA synthesis were determined for the indicated genes at 9 hpi using Click-iT nascent RNA labeling and plotted as fold change in PR8-PA(fs) infected cells vs. PR8 wild-type infected cells. 18S values were normalized to input RNA concentration, whereas viral mRNA values were normalized to 18S levels. Error bars = st.dev. from three independent biological replicates. *,** = *p* value < 0.05, 0.01 for PR8-PA(fs)- vs. PR8-infected cells.

When determining the titers of recombinant PR8-PA(fs) virus stocks generated in our laboratory, we consistently observed reduction in plaque size compared to the parental wild-type PR8 strain (Fig 5A). This suggests that PA-X function is important for efficient multi-round replication of IAV in culture, consistent with previous reports [19]. However, to date the exact contribution of PA-X to virus fitness remains poorly understood. In order to compare viral RNA and protein production between the wild-type PR8 virus and the PA-X deficient PR8-PA(fs) strain, we infected A549 cells with the same number of virions and collected parallel samples for immunofluorescence staining, western blotting for viral proteins, and total RNA isolation. Immunofluorescence staining for the viral PA protein confirmed that in our experiments a similar number of cells were infected with either wild-type PR8 or the mutant PR8-PA(fs) virus (Fig 5B). Similar PA protein accumulation was also detected in wild-type and mutant virus-infected cell lysates at multiple time points (Fig 5C). However, the accumulation of the viral proteins M1, NS1, and especially M2 was significantly slower in cells infected with PR8-PA(fs) mutant virus compared to wild-type virus-infected cells (Fig 5C and 5D), which may be the cause for the reduction in plaque size. The reduced viral protein accumulation in the absence of PA-X was unexpected; if PA-X were able to degrade viral RNAs, we would expect an increase in viral protein levels in PR8-PA(fs) infected cells. These findings suggest potential secondary consequences of PA-X-mediated RNA degradation on viral protein accumulation. Importantly, we saw comparable levels of most viral mRNAs and genomic vRNAs in PR8 and PR8-PA(fs) infected cells (Fig 5E). Only M1 mRNA accumulated to significantly higher levels in PR8-PA(fs) infected cells at later time points, with roughly 1.6-times more M1 transcript at 12 and 15 hpi (Fig 5E and 5F). M2 and NEP mRNAs, which are generated through splicing of M1 and NS1 transcripts respectively, were slightly reduced in PR8-PA(fs) infected cells (Fig 5E and 5F). Metabolic pulse-labeling of nascent RNAs by Click-IT chemistry revealed that the changes in M1 and NEP total mRNA levels resulted from increased (M1) or decreased (NEP) synthesis rates, with relative total mRNA levels at 12 hpi overall matching the altered rates of synthesis at 9 hpi (Fig 5F and 5G). Taken together, these data show that unlike cellular mRNAs synthesized by host Pol II, RdRp-generated viral mRNAs are not subject to PA-X mediated degradation. In fact, viral RdRp-generated mRNAs are translated more efficiently in the presence of PA-X, which may be due to reduced competition with host mRNAs for access to translation machinery.

### Alterations in mRNA 3’ end processing prevent PA-X mediated degradation

**Fig 6:**
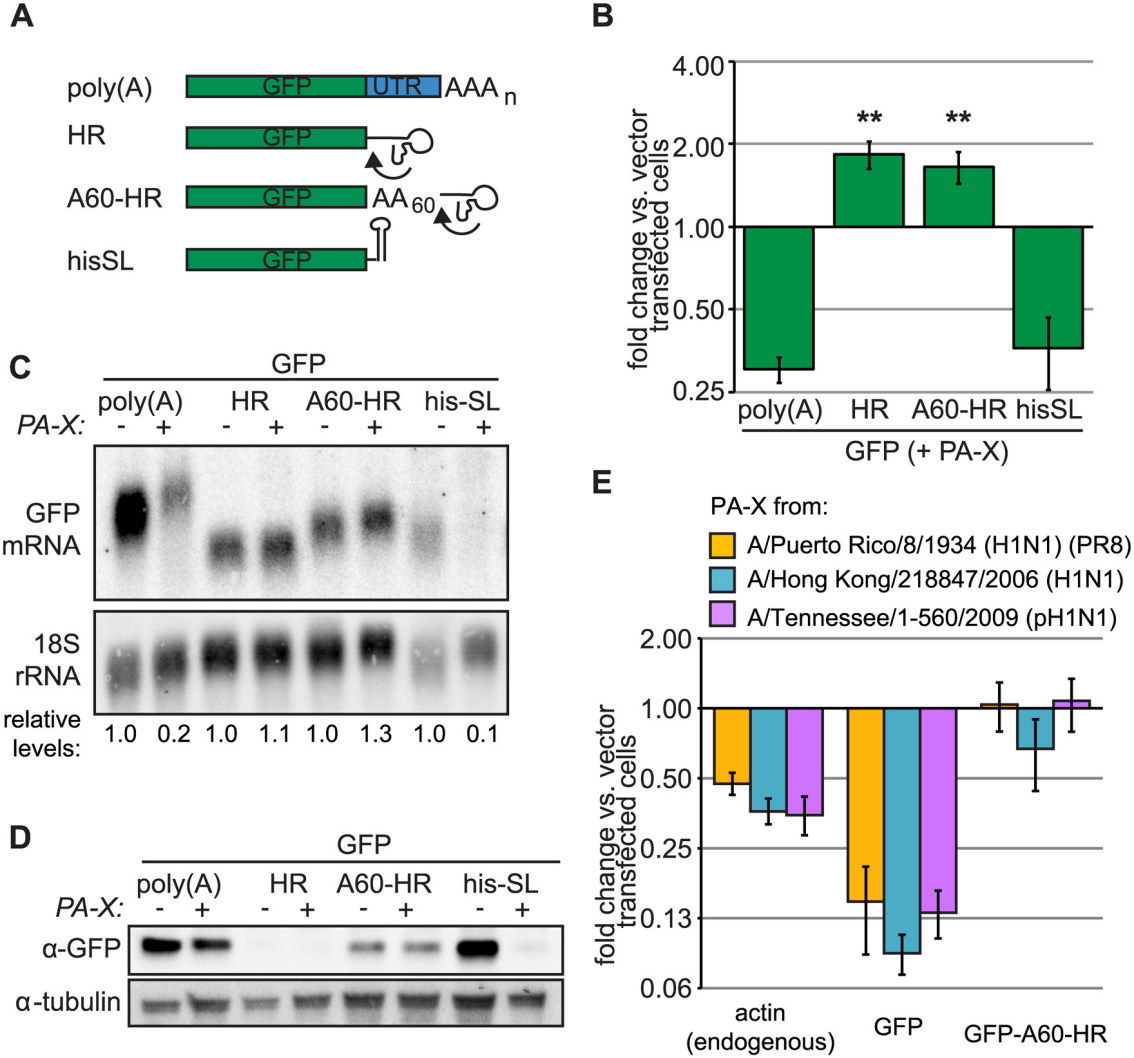
Canonical processing of the 3’ end of Pol II transcripts confers sensitivity to PA-X. (A) Diagram of the GFP reporter constructs used in the figure, which bear different 3’ mRNA ends: the canonical poly(A) tail added by cellular processing machinery (poly(A)), a hammerhead ribozyme (HR), the hammerhead ribozyme proceeded by a 60-nt templated poly(A) stretch (A60-HR), or the histone stem-loop structure (hisSL). The arrows indicate where the HR self-cleaves the RNA. (B-D) HEK 293Ts were transfected with indicated Pol-II driven GFP reporters and either PA-X or empty vector. Total cellular RNA was extracted and GFP mRNA levels were measured by RT-qPCR (B) or northern blotting (C). GFP expression normalized by 18S rRNA abundance is plotted (B) or reported (C, relative levels) as fold change in PA-X-expressing vs. vector-transfected cells. (D) Levels of GFP for experiments in panel B were assessed by western blot, using tubulin as a loading control. A representative image is shown. ** = *p* value (Student’s *t*-test) < 0.01 for fold change of indicated reporter vs. fold change of poly(A) GFP. (E) HEK 293Ts were transfected with indicated Pol-II driven GFP reporters and PA-X from three different IAV strains or empty vector. Total cellular RNA was extracted and GFP or actin mRNA levels were measured by RT-qPCR. GFP expression normalized by 18S rRNA abundance is plotted as fold change in PA-X-expressing vs. vector-transfected cells. Error bars = s.e.m.

The selectivity of PA-X for Pol II transcripts could be linked to the RNA polymerase directly, or to processing events that are specific to Pol II transcripts. In particular, termination of transcription by Pol II is normally followed by addition of a non-templated poly(A) tail at the 3’ end of the RNA, which is absent from Pol I and Pol III transcripts, and the canonical polyadenylation signal serves to direct termination, cleavage of the RNA and polyadenylation. An alternative stem loop termination signal is used for histone mRNAs [42], whereas some ncRNAs have distinct 3’ end processing mechanisms [43,44]. To test whether PA-X targeting of host Pol II transcripts is coupled to canonical 3’-end processing, we used a set of constructs that had altered 3’ ends. The 3’ polyadenylation signal and the 3’ untranslated region (UTR) of the GFP reporter were replaced by a self-cleaving hammerhead ribozyme (HR) or the histone stem loop termination region (hisSL) (Fig 6A) [45]. The HR sequence obviated the need for the cleavage and polyadenylation machinery in truncating the RNA 3’ end. We found that PA-X was unable to degrade the GFP-HR construct (Fig 6B and 6C), which was also not translated as previously reported (Fig 6D). Addition of a 60 nt templated stretch of adenosines that mimicked a poly(A) tail (GFP-A60-HR) restored translation of the GFP-HR construct (Fig 6D) while GFP-A60-HR mRNA levels were still unaffected by PA-X (Fig 6B and 6C), suggesting that the lack of cellular 3’ end processing of this RNA prevented targeting by PA-X. By contrast, replacement of the 3’ poly(A) signal with a histone stem loop termination region promoted down-regulation by PA-X (Fig 6B and 6C), consistent with the fact that the histone HIST1H3C mRNA was also down-regulated by PA-X expression (Fig 1B and 1C, S1A and S1B Fig). Northern blotting confirmed that the size of the GFP RNA species is consistent with the expected processing route (Fig 6C). In addition, we found that similarly to PR8 PA-X, PA-X variants from two other human strains of IAV, a pre-pandemic 2006 H1N1 strain and a 2009 pandemic H1N1 strain could degrade endogenous actin mRNA and transfected poly(A)-tailed GFP mRNA, but not GFP-A60-HR mRNA (Fig 6E). Previous examples of protection of specific RNAs from host shutoff RNases have focused on the presence of protective elements. This is the case for SARS CoV mRNAs, which are protected from nsp1-mediated degradation [46] or for the host IL-6 mRNA during KSHV infection, which is protected from SOX-mediated degradation through 3’ UTR elements [47,48]. However, our results strongly suggest that resistance of IAV mRNAs to PA-X is due to their differential biogenesis pathway, in particular the 3’ maturation mechanism. These results, together with our viral mRNA data (Fig 5E and 5F), suggest that the process of polyadenylation or the presence of the poly(A) tail *per se* are not sufficient or necessary for PA-X targeting of mRNAs. Instead they hint at a more universal feature of the 3’ end processing of the Pol II transcribed RNAs that earmark target RNAs for PA-X mediated cleavage. Since mRNA processing and maturation are linked to nuclear export, we compared the PA-X mediated changes in reporter transcript levels between cytoplasmic and nuclear RNA fractions. Importantly, PA-X was able to target both the cytoplasmic and the nuclear pools of the susceptible reporter RNAs (poly(A)-tailed GFP and GFP-hisL), while we failed to detect down-regulation of the HR bearing constructs in either fraction (S3A Fig). This demonstrates that PA-X is able to function in the nucleus and target transcripts prior to their export in the cytoplasm.

### Nuclear accumulation of PA-X is mediated by the C-terminal X-ORF and correlates with shutoff function

**Fig 7:**
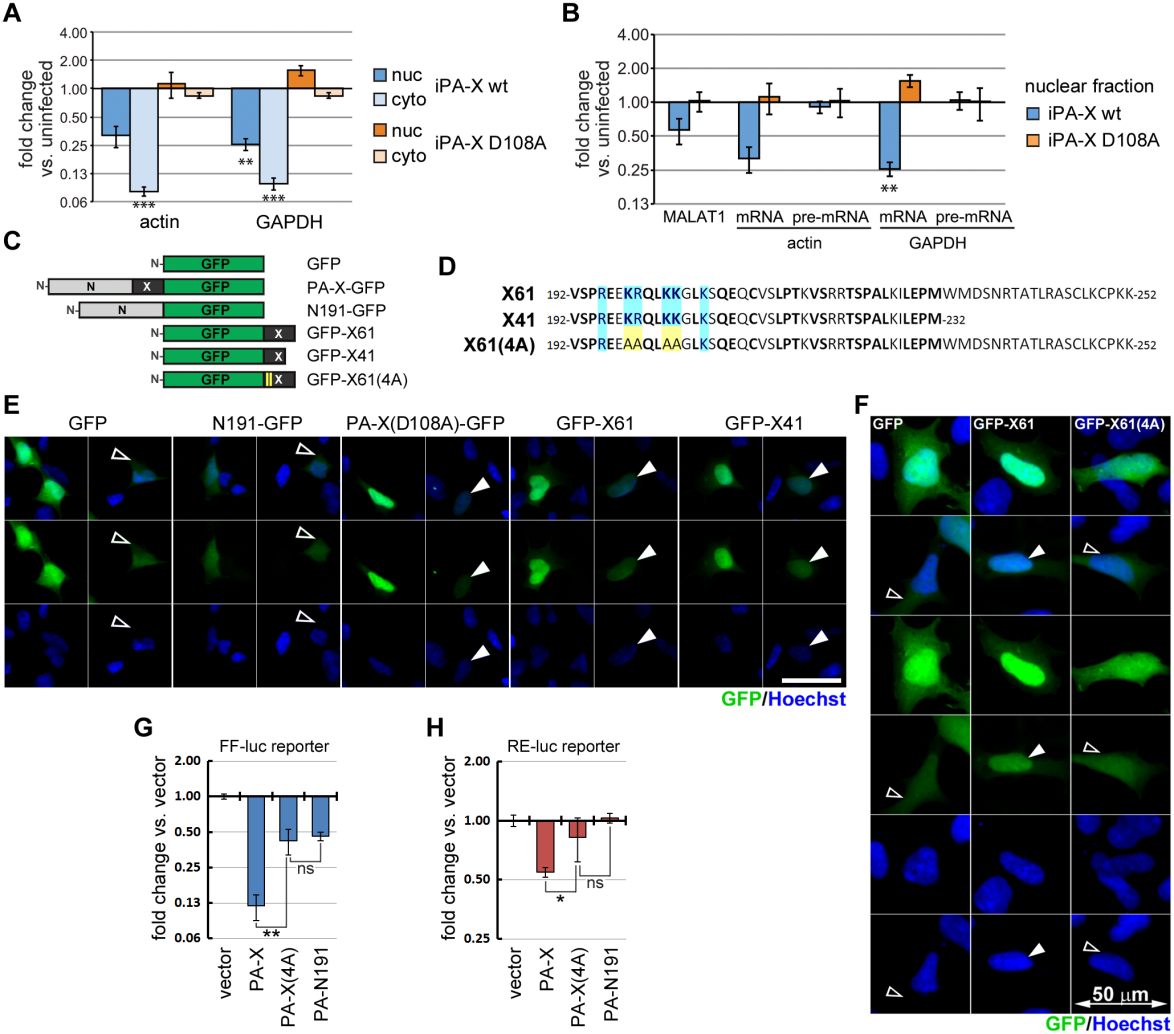
PA-X accumulates and functions in the cell nucleus. (A and B) A549 cells expressing doxycycline-inducible wild-type PA-X-myc (“iPA-X wt”, line #10) or a catalytically inactive mutant (“iPA-X D108A”, line #8) were treated with doxycycline for 18 h to induce expression of PA-X. Total RNA was isolated from either nuclear or cytoplasmic fraction and RT-qPCR was performed to measure levels of the indicated endogenous RNAs. RNA levels were normalized by 18S rRNA levels and are plotted as fold change in induced (PA-X-expressing) vs. uninduced cells. In (A), target level changes in nuclear (nuc) and cytoplasmic (cyto) fractions are presented. In (B), changes in total nuclear mRNA and in unspliced pre-mRNA are compared. Error bars = s.e.m, n = 3–4. (C) Schematic diagram of the GFP fusion constructs generated in this study. (D) Amino acid sequences of the X-ORF from PR8 strain (X61) aligned to a shortened 41 amino-acid construct (X41) and the mutant X-ORF with alanine substitutions at four basic residues (X61(4A)). Amino acids that are highly conserved between IAV strains are shown in bold and the basic residues that are important for PA-X function are highlighted in blue. (E and F) Fluorescence microscopy images of 293A cells transfected with the expression constructs for the indicated GFP fusion proteins listed in (C). Two fields of view are shown for each transfection experiment. Representative cells with nuclear GFP signal accumulation are highlighted with filled arrowheads and those that have even signal distribution with open arrowheads. Nuclei were stained with Hoechst dye. (G and H) Relative Firefly (FF-luc, G) and Renilla (RE-luc, H) luciferase reporter expression in 293A cells co-transfected with these reporters and the indicated wild-type and mutant PA-X constructs. Error bars = st. dev. from four independent biological replicates. *,** = *p* value < 0.05, 0.01 (**); ns = *p* value > 0.05 (Anova single factor).

Although all other host shutoff RNases are proposed to work in the cytoplasm, our results link PA-X targeting to 3’ end processing (Fig 6) and show degradation of reporter transcripts in the nucleus (S3A Fig). This suggests that PA-X may degrade nascent RNAs shortly after transcription. To test this hypothesis, we measured changes in the levels of endogenous transcripts in the nuclear and the cytoplasmic RNA fractions upon PA-X induction in A549 iPA-X cells (Fig 7A). Consistent with the reporter RNA data, both nuclear and cytoplasmic actin and GAPDH mRNAs were down-regulated (Fig 7A). Next, we compared PA-X dependent decrease in the levels of unspliced pre-mRNAs and mature mRNAs for these genes, as well as the MALAT1 ncRNA, in the nuclear fraction. We found that unlike processed mRNAs and MALAT1, pre-mRNAs were not down-regulated (Fig 7B). Because most splicing is thought to occur cotranscriptionally [49], unspliced RNA levels likely reflect nascent RNA levels. Thus, this result reaffirms our finding that a later step of processing specific to Pol II transcripts is crucial for targeting by PA-X. Previous studies have demonstrated that the C-terminal domain of PA-X created by the frameshift, the X-ORF, is important for PA-X shutoff activity [34,50]. In particular the recent study by Oishi *et al*. [34] highlighted the importance of 6 highly conserved basic residues within the first 15 amino acids of X-ORF for PA-X function. Since we demonstrated that PA-X can function in the nucleus, we set out to examine the subcellular localization of PA-X and the effect of the X-ORF on the nucleocytoplasmic distribution of this protein. To this end we constructed a series of GFP fusion constructs containing the full-length wild type (PA-X-GFP) or catalytic mutant PA-X protein (PA-X(D108A)-GFP), the N-terminal nuclease domain of PA-X (PA-N191-GFP), the X-ORF (GFP-X61) or shortened version of the X-ORF that mimics variants of PA-X with a 41 amino acid tail (GFP-X41, Fig 7C and 7D). In addition, we created a GFP X-ORF fusion protein in which four out of the six functionally important basic residues were mutated to alanines (GFP-X61(4A), Fig 7C and 7D). We observed that GFP-tagged PA-X and all fusion proteins containing the first 41 amino acids of X-ORF, although found throughout the cell, concentrated in the nucleus (Fig 7E, S4A Fig). By contrast, the subcellular distribution of PA-N191-GFP and GFP-X61(4A) was highly similar to that of GFP alone (Fig 7E and 7F and S4A Fig). Although GFP is small enough to show some nuclear accumulation on its own, the nuclear accumulation of the X-ORF fusion proteins was much more robust, and analysis of cells that expressed lower levels of the fusion proteins showed almost exclusive nuclear accumulation of the protein (Fig 7E and S4A Fig). PA-X-GFP was efficient at blocking expression of co-transfected luciferase reporter constructs despite the large tag (S4B and S4C Fig). Moreover, consistent with previous reports, the lack of the X-ORF severely impaired host-shutoff activity (S4B and S4C Fig, PA-N191-GFP). As expected, despite nuclear accumulation, the PA-X(D108A)-GFP mutant, GFP-X61 or GFP-X41 did not possess the shutoff activity due to lack of the functional nuclease domain (S4B and S4C Fig), although a previous report has argued that overexpression of X-ORF peptides alone has effects on gene expression [51]. Finally, we compared the shutoff function of myc-tagged constructs of the wild type PA-X, a PA-X with the four K/R-to-A mutations in the X-ORF (PA-X(4A)), and the N-terminal nuclease domain alone (Fig 7G and 7H) using luciferase reporter assay. In this assay, PA-X no longer inhibited luciferase expression when the four basic residues required for the nuclear localization function of the X-ORF (Fig 7F) were mutated to alanine, similarly to the complete deletion of the X-ORF (Fig 7G and 7H). Collectively these data reveal a striking correlation between nuclear accumulation of the PA-X-GFP fusion proteins and their shutoff activity. More specifically, it shows that X-ORF acts as a nuclear accumulation domain and that the conserved basic residues important for PA-X shutoff function participate in key molecular interactions governing X-ORF-mediated nuclear accumulation.

## Discussion

Our study provides new mechanistic insights into the specificity of PA-X, the most recently identified viral host shutoff nuclease. We demonstrate here for the first time that PA-X is recruited to the nucleus, selectively targets a subset of host transcripts, and is not active against viral mRNAs. Moreover, we uncover a novel route of target discrimination by a viral RNase, which takes advantage of the divergent mRNA biogenesis mechanisms that generate viral and host transcripts. We demonstrate that PA-X shares some specificity features with other host shutoff nucleases, as it selectively targets products of cellular RNA Pol II, while sparing Pol I and Pol III transcripts, and requires host RNases to complete RNA degradation. Interestingly, the mechanism for PA-X targeting of Pol II transcripts is not related to the translatability of the mRNAs. Instead, PA-X targeting is directly linked to synthesis by RNA Pol II complex or early processing events unique to Pol II transcripts. Moreover, we find that PA-X likely degrades RNAs in the nucleus, because it accumulates in this compartment and affects both the nuclear and cytoplasmic fraction of its target RNAs. Both the host shutoff activity of PA-X and the nuclear localization function of the C-terminal X-ORF are dependent on the presence of a set of basic residues in the X-ORF, indicating a correlation between nuclear localization and RNA targeting. Thus, the mechanism of PA-X targeting may be different from other shutoff RNases because it is tightly linked to the mechanism of biogenesis of host and viral mRNAs in the nucleus.

Like other host shutoff endonucleases SOX, vhs and nsp1 [22,29,37], PA-X carries out an initial cleavage of its targets and then relies on host enzymes to complete degradation of RNA fragments. Nonetheless, there are key differences between PA-X and these previously described enzymes. PA-X cleavage is not directed by specific sequence elements, as previously reported for the gamma-herpesviral host-shutoff RNases [14,15,29], or by proximity to the 5’ end of the message, like SARS CoV nsp1 [22] or alphaherpesvirus vhs [37]. Analysis of the GFP reporter upon Xrn1 knock-down suggests that PA-X cuts RNAs throughout the transcript (Fig 2D). Whether this is true for endogenous mRNA targets remains to be determined. Moreover, whereas a tight relationship with translational machinery has been reported for some of the endonucleases (vhs, nsp1 [22,30,31]), we find that translation is not a key determinant of PA-X targeting. Pol II-transcribed RNAs that are not translated, like the 5’ hairpin containing GFP reporter and the endogenous ncRNAs MALAT1 or TP53TG1 are down-regulated by PA-X (Fig 3A, 3C and 3D, S2A, S2C–S2E Fig). By contrast, translated viral mRNAs (Fig 5C–5F) and the translated GFP-A60-HR and T7-polymerase driven luciferase reporter constructs (Figs 3B, 6B–6E, S2B and S3A Figs) are not affected.

Although we cannot exclude that other processes connected to translation are important for RNA degradation, our data strongly indicate that translation *per se* is not required. Our data point instead to the processing of Pol II transcripts in the nucleus as the link between PA-X and its target. Importantly, our examination of endogenous and reporter Pol II transcripts that differ in their 3’ end processing suggests that this step in biogenesis may be recognised by PA-X to find its targets. In our experiments, GFP reporters bearing the canonical polyadenylation signal (PAS) or a histone stem loop at the 3’ end were efficiently inhibited by PA-X. By contrast, the same reporter with a templated poly(A) stretch followed by a self-cleaving hammerhead ribozyme sequence was not degraded by PA-X despite being translated (Fig 6). Replication-dependent histone mRNAs and polyadenylated mRNAs are processed very differently, but some proteins participate in both mechanisms including the scaffold protein symplekin and the cleavage and polyadenylation specificity factor (CPSF) complex subunit CPSF-73 [52,53]. Nuclear localisation and targeting of nuclear mRNA pools by PA-X demonstrated in our study also strongly hint at a mechanism that involves association with target mRNAs prior to their engagement in translation in the cytoplasm. The fact that in our experiments nuclear unspliced pre-mRNAs were largely unaffected by PA-X (Fig 7B) also suggests that the later stages of mRNA maturation are responsible for PA-X recruitment. A host protein involved in Pol II transcript 3’ end processing could in theory earmark the RNA for cleavage by PA-X, although our data on the nuclear MALAT1 ncRNA hints at a more complex model. The MALAT1 gene includes a canonical PAS, but the most abundant MALAT1 transcript is processed by RNase P at a location upstream of the PAS, and is therefore not polyadenylated [43]. It is possible that the presence of the PAS could still promote co-transcriptional recruitment of cellular factors necessary for PA-X targeting, regardless of whether it is used for 3’-end processing, because the PAS can direct transcription termination independently of 3’ cleavage and polyadenylation [54]. In general, determining whether PA-X physically interacts with transcription and RNA processing machinery will be key to understanding how PA-X targets Pol II-transcribed RNAs in the nucleus. A major challenge for future studies will be to disentangle the highly interconnected steps of RNA metabolism to better understand PA-X function.

Our data also demonstrate a new cellular function for the C-terminal X-ORF, whose required function in host shutoff [34,50] is poorly understood. We found that the conserved 41-amino acid portion of the X-ORF is sufficient to cause nuclear accumulation of GFP fusion proteins and acts as a nuclear targeting sequence (Fig 7C–7F and S4A Fig). Moreover, nuclear localization of PA-X is tightly linked to reporter shutoff efficiency (Fig 7G and 7H). At present it remains to be determined whether the X-ORF contains a functional NLS or whether it causes nuclear accumulation through interactions with other NLS-containing proteins. The latter mechanism appears more likely, because the region responsible for the nuclear localization function of X-ORF lacks homology to well-characterized NLS consensus sequences (Fig 7D). Also, because increased *in vitro* activity has been reported for PA-X vs. the N terminal RNase domain alone [38], the X-ORF probably has additional roles in RNA degradation by PA-X.

The fate of viral mRNAs and proteins during host shutoff has been a topic of intense study, as the expectation is that viral products should be protected from host shutoff to confer a selective advantage. For example, SARS CoV transcripts are protected from RNA degradation by nsp1 by a common 5’ leader sequence [46]. Interestingly, some viruses, like gamma-herpesviruses, have no mechanism for widespread protection of viral mRNAs [55]. Early studies of IAV host shutoff demonstrated that the virus selectively inhibits translation of host mRNAs, while IAV mRNAs appear resistant [1]. One of the well characterised host shutoff mechanisms employed by IAV is mediated by the NS1 protein from human IAV strains that binds and inactivates cellular CPSF30, preventing polyadenylation and nuclear export of host pre-mRNAs [2]. IAV mRNAs are exempt from CPSF30 inactivation because they rely exclusively on RdRp for addition of the poly(A) tail. Remarkably, we discovered that mRNA 3’ end processing in the nucleus may serve as the basis for the protection of viral mRNAs from PA-X as well. Future studies will determine whether similarly to NS1, PA-X is recruited to its target RNAs through direct interaction with one of the subunits of 3’ end processing machinery.

We observed no major effects of PA-X on the levels of viral mRNAs and vRNAs comparing viral RNA levels between wild-type and PA-X deficient mutant viruses (Fig 5E). The only exceptions are the M1 mRNA, which is transcriptionally up-regulated, albeit to a small degree, and the spliced M2 and NEP mRNAs, which are synthesized at slightly lower rates (Fig 5E–5G). One recent study reported an increase in RdRp activity and levels of PA protein in PA-X-deficient viruses [17]. We note that we do not see an increase in PA levels in the PR8-PA(fs) mutant virus-infected cells (Fig 5B–5D). In fact, several of the IAV PR8 proteins accumulated to lower levels in the absence of PA-X, including M1, despite an increase in its mRNA levels. This suggests that, like HSV-1 vhs [56], PA-X may also function to reduce competition for translational machinery between host and viral mRNAs. In turn, changes in viral protein accumulation could explain the transcriptional and splicing changes we detected, because viral proteins have roles in regulating viral gene expression. For example, NS1 is required for the production of correct levels of spliced M2 mRNA [57], and we detect both changes in NS1 protein accumulation and in M2 mRNA and protein levels. The reduced accumulation of M2 in particular may be responsible for the small plaque phenotype that we have observed with PR8-PA(fs) virus, as mutations in M2 have been reported to give rise to a small plaque phenotype [58]. Previous studies using different strains of IAV have reached different conclusions on the role of PA-X in replication of the virus in tissue culture and virulence in animal models [5,16–19], suggesting that the effect of PA-X on viral replication and disease is strain dependent. In this study we used the mouse adapted PR8 strain that possesses NS1 protein lacking the ability to bind CPSF30 and block host mRNA polyadenylation. Because PR8 lacks at least one of the other IAV host-shutoff mechanisms, PA-X could play a more important role in host shutoff and/or have stronger effect on viral fitness in our model. Additional studies examining the interplay of the PA-X and NS1-mediated host shutoff mechanisms will further advance our understanding of IAV host shutoff and its role in viral replication, pathogenicity, and species adaptation.

## Materials and Methods

### Plasmids

pCR3.1-PA-X-myc and pCR3.1-PA-N191-myc were generated by inserting the PCR-amplified full length PA-X or the PA nuclease domain sequences from pCR3.1-PA-X plasmid [41] into the pCR3.1-myc vector [59] between KpnI and MluI sites. Subsequently, pCR3.1-PA-X(D108A)-myc and pCR3.1-PA-X(4A)-myc vectors were generated by site-directed mutagenesis of the pCR3.1-PA-X-myc plasmid. In order to create the GFP-tagged constructs pCR3.1-PA-X-GFP, pCR3.1-PA-X(D108A)-GFP, and pCR3.1-PA-N191-GFP, the myc tag sequence flanked by MluI and XhoI sites was replaced with the PCR-amplified EGFP ORF. pCR3.1-EGFP control vector was created by inserting EGFP ORF between BamHI and XhoI sites of pCR3.1-myc plasmid. An AgeI restriction site was introduced by PCR immediately upstream of the EGFP ORF stop codon to enable insertion of the C-terminal X-ORF sequences. Subsequently, full-length (X61), shortened (X41), and mutant [X61(4A)] X-ORF sequences were PCR-amplified from the pCR3.1-PA-X-myc and pCR3.1-PA-X(4A)-myc templates without the inclusion of the myc tag and inserted between AgeI and XhoI sites to create pCR3.1-GFP-X61, pCR3.1-GFP-X41, and pCR3.1-GFP-X61(4A) vectors. pCR3.1–2006 H1N1 PA-X-myc and pCR3.1–2009 TN H1N1 PA-X-myc were generated by subcloning PA-X (including the 5’ UTR) from A/Hong Kong/218847/2006 (H1N1) and A/Tennessee/1-560/2009 (pandemic H1N1) IAV segment 3 constructs that were a kind gift from R. Webby (St Jude’s Children Research Hospital, Memphis, TN) into the SalI and MluI sites of pCR3.1-myc. A single nucleotide was deleted to obtain the PA-X coding sequence using overlap extension. pd2-eGFP-N1 was purchased from Clontech. pd2-eGFP-N1-CMVd1 (used in Fig 1A) was generated by deleting 457 bp from the CMV IE promoter in pd2-eGFP-N1, in order to reduce the constitutive levels of GFP mRNA and protein. GFP-3’SLII, GFP-codingSLII, GFP-HR, GFP-A60-HR, GFP-hisSL and hp-GFP constructs are based on pd2-eGFP-N1 and were previously described [14,29,45]. Pol I GFP, Pol III GFP, pCDEF3-SOX, pCAGGS-nsp1, and pCDNA3.1-DsRedExpress-DR were previously described [8,14,29]. pTRIPZ-shNS and pTRIPZ-Xrn1 were purchased from Thermoscientific (shXrn1: clone V2THS_89028/RHS4696-99704634, targeting sequence: TATGGTGAGATATACTATG). pTRIPZ-PA-X-myc was generated by replacing the RFP sequence in pTRIPZ-shNS with the PA-X-myc coding sequence using the AgeI and ClaI restriction sites. pTRIPZ-RFP-SV40-3’UTR was generated by substituting the shRNA sequence downstream of the RFP coding sequence in pTRIPZ-shNS with the SV40 3’ UTR from pd2-eGFP-N1. The RFP sequence was then replaced with PA-X-myc or PA-X(D108A)-myc to generate pTRIPZ-PA-X-myc-SV40-3’UTR and pTRIPZ-PA-X(D108A)-myc-SV40-3’UTR using the AgeI and ClaI restriction sites. All PA-X constructs also include the viral 5’ UTR of PR8 IAV segment 3. The T7 polymerase and T7-driven EMCV-luciferase constructs [40] were a kind gift from E. Heldwein (Tufts University School of Medicine, Boston, MA). They were co-transfected in 293T iPA-X cells to obtain luciferase transcription by T7 polymerase. Luciferase RNA and luciferase activity were only detected when the T7 polymerase expression construct was co-transfected, confirming that the luciferase RNA is solely transcribed by T7. pTRE2-Fluc and pTRE2-Rluc vectors were previously described [60]. pGL4.32 and pGL4.74 reporter plasmids were purchased from Promega. Sequence information for the vectors generated in this study is available upon request.

### Cell lines, transfections and luciferase reporter assays

Mouse embryonic fibroblasts (gift from Dr. Kedersha, Brigham and Women’s Hospital, Boston, MA), HeLa Tet-Off (Clontech), HEK 293A (ATCC), HEK 293T (ATCC), A549 (ATCC), and their derivative 293T and A549 “iPA-X” cells were maintained in Dulbecco’s modified Eagle’s medium (DMEM; high glucose, Life Technologies) supplemented with 10% fetal bovine serum (FBS, Hyclone) at 37°C in 5% CO_2_ atmosphere. HEK 293T iPA-X cells were generated by lentiviral transduction using pTRIPZ-PA-X-myc. A549 iPA-X were generated by lentiviral transduction using pTRIPZ-PA-X-myc-SV40-3’UTR or pTRIPZ-PA-X(D108A)-myc-SV40-3’UTR. Clonal populations were selected and several lines were used for all experiments. PA-X expression was induced by addition of doxycycline (Fisher, 0.2–1 μg/ml final concentration depending on the cell line) for approximately 18 h prior to harvesting, with the exception of half-life experiments, in which doxycycline was added for 4.5 h before actinomycin D addition. HEK 293T shNS (also referred to as “iRFP” cells) and shXrn1 cells [15] were generated using pTRIPZ-shNS (Thermo scientific) and pTRIPZ-shXrn1 (Thermo scientific) respectively. To induce expression of the shRNAs, cells were treated with 1 μg/ml doxycycline for 4–5 days prior to harvesting. For experiments using reporter constructs, 800 ng of DNA was transfected in 12-well plate wells using polyethylenimine (Fisher). For the experiment in Fig 6E, FugeneHD (Promega) was used for transfection, in order to achieve high transfection efficiency (estimated at > 75% based on GFP fluorescence). RNA and proteins were harvested as detailed below 1 day after transfection. For measuring the levels of the Firefly and Renilla luciferase reporter expression the Dual-Luciferase Reporter Assay Kit (Promega) was used according to manufacturer protocol.

### Virus infections

The A/PuertoRico/8/34/(H1N1) (PR8) and the recombinant mutant PR8-PA(fs) viruses are described in [41]. Virus stocks used for experiments were produced and titrated by plaque assays as described [41]. Two independent recombinant virus rescues were performed as described in [61] for both wild-type PR8 and PR8-PA(fs) and used in experimental infection replicates. The genomic RNA segment 3 of all virus stocks was verified by sequencing. For each infection, after 1-hour inoculation with virus dilutions, cells were washed with PBS and cultured in infection medium (0.5% bovine serum albumin (BSA) in DMEM) and incubated at 37°C in 5% CO2 atmosphere.

### Immunofluorescence staining

Cells grown on glass coverslips were fixed and immunostained according to the protocol in [62] using mouse monoclonal antibody to PABP1 (sc-32318, Santa Cruz Biotechnology); goat polyclonal antibody to influenza virus (ab20841, Abcam), or rabbit antibody to PA (GeneTex-125932) at manufacturer-recommended dilutions. Nuclei were stained with Hoechst dye (Invitrogen). AlexaFluor-conjugated secondary antibodies (Molecular Probes) were used at 1:1,000 dilution. Images were captured using Zeiss Axioplan II microscope.

### RNA extraction, fractionation, northern blotting and RT-qPCR

For steady-state measurements of RNA levels in cells ectopically expressing PA-X protein, RNA was harvested 1 day after transfection or 18 h after PA-X induction in iPA-X cells. For half-life measurements, PA-X (wt or D108A) was induced in A549 iPA-X cells for 4.5 h, followed by addition of actinomycin D at 10 μg/ml final concentration. RNA was collected 0, 2, 4, 6 h after actinomycin D addition, as well as prior to doxycycline treatment.

RNA for Northern blotting was harvested and extracted using Trizol reagent (Life Technologies) following manufacturer’s protocol. To isolate cytoplasmic vs. nuclear fractions, cells were lysed in 0.1% NP-40 in Dulbecco’s phosphate buffered saline (DPBS) and the nuclear pellet was spun down. The supernatant was collected as the cytoplasmic fraction. The pellet was washed in 0.1% NP-40 in DPBS, then it was collected as the nuclear fraction. The RNA was extracted from both fractions using Trizol (Life Technologies) following manufacturer’s protocol.

The RNA for northern blotting was run on a 1.8% agarose/formaldehyde gel and transferred by capillary action onto nitrocellulose membrane (Bio-Rad) using 10x SSC buffer. Northern blots were probed with probes against the SV40 3’ UTR present in pd2-eGFP-N1 [29], the first 450 nt of the GFP coding sequence, the endogenous 18S rRNA [45], 7SL and U2 ncRNAs in Church buffer. Probes were generated and radiolabeled using the Decaprime II kit (Life Technologies). Blots were imaged using a Fujifilm scanner FLA-9000. Quantification of the blots was carried out using ImageJ [63]. Figures show images representative of multiple biological replicates.

Total cellular RNA for RT-qPCR in iPA-X and transfected cells was harvested and isolated using Zymo mini-prep kit (Zymo Research) following manufacturer’s protocol. cDNA was generated using iScript (Bio-Rad) from DNase-treated RNA.

For analyses of mRNA and vRNA levels in A549 cells infected with IAV, total RNA was isolated at 6, 9, 12, and 15 h post-infection using Qiagen RNeasy Plus kit. For cDNA synthesis, Thermo Maxima H Minus kit was used with gene-specific primer for 18S rRNA combined with either oligo(dT) (for mRNA targets) or influenza vRNA-specific Uni12 primer [64]. To minimize primer-less reverse transcription, after primer annealing reactions were carried out at 65°C according to manufacturers’ protocol.

Quantitative PCR analysis was performed using iTaq Universal SYBR Green Supermix (Bio-Rad) and Ct values were analyzed using the BioRad CFX Connect Real-Time System qPCR and Bio-Rad CFX Manager 3.1 program analysis. S1 Table lists primers used for qPCR. Pre-mRNA measurements were carried out with primer sets that have been previously used in the literature and that are located within predicted introns [65]. All qPCR experiments shown are the average of three or more biological replicates. Within each biological replicate, RNA levels were assessed using the average of at least two technical replicates. One- and two-sample Student’s *t*-test was used to analyze values for significant differences.

### Click-iT RNA labeling

Metabolic labeling and isolation of nascent RNA in A549 cells infected with wild-type PR8 or mutant PR8-PA(fs) viruses was performed using Molecular Probes Click-iT Nascent RNA Capture Kit. At 8 hpi, 0.4 mM Click-iT nucleotide analogue 5-ethynyl uridine (EU) was added to the infection media for 1 hr at 37°C. At 9 hpi monolayers were washed twice with PBS and total RNA was isolated as described using Qiagen RNeasy Plus kit. Biotinylation and subsequent purification of EU-labelled RNA was performed according to the kit manufacturer protocol. cDNA synthesis and qPCR was performed on streptavidin beads as directed by the kit manufacturer protocol and described in methods section above.

### Protein extraction, western blotting and antibodies

Total cellular protein was collected in protein lysis buffer (10 mM Tris pH 8, 150 mM NaCl, 1% Triton x100, and cOmplete EDTA-free protease inhibitor cocktail (Roche)) unless specified otherwise in the text. Proteins were separated on SDS-PAGE gels and transferred onto PVDF membranes (Millipore). Western blots were performed in PBST with 5% milk or TBST with 4% bovine serum albumin. The following antibodies were used: Xrn1 (1:200, Santa Cruz-16598), IAV NS1 (1:1,000, gift from Kevin Coombs [66], clone 8C7) M2 (1:1,000, Abcam ab5416), GFP (1:500, Santa Cruz-8334), β tubulin (1:200, Santa Cruz-9104), actin (1:4,000, HRP-conjugated, Cell Signalling #5125), IAV PA (GeneTex-125932), IAV (1:2,000, Abcam ab20841, recognizes NP, M1, and (weakly) HA proteins of PR8 strain). Secondary antibodies were purchased from Southern BioTech (rabbit, mouse) or Santa Cruz (goat) and used at 1:3,000 to 1:5,000 dilution.

### Accession numbers (NCBI Gene symbol/NCBI gene ID)

Human genes: β-actin: ACTB/60; β-tubulin: TUBB/ 203068; EEF1A: EEF1A1/1915; Histone cluster 1 H3C: HIST1H3C/8352; POLR2A: POLR2A/5430; MALAT1: MALAT1/378938; TP53TG1: TP53TG1/11257; GAPDH: GAPDH/2597; GUSB: GUSB/2990; RPS6: RPS6/6194; RPS18: RPS18/6222; 7SL: RN7SL1/6029; 7SK: RN7SK/125050; U2: RNU2-1/6066.

IAV genes: PR8 PA-X: PA-X/13229134.

## Supporting Information

S1 Fig RNA targeting by PA-X is dependent on the host RNA polymerase complex that transcribes the RNA (additional data from independently derived cell lines). (A-C) HEK 293T (A) or A549 (B-C) cells expressing doxycycline-inducible wild-type PA-X-myc (“iPA-X wt”), a catalytically inactive mutant (“iPA-X D108A”) or RFP (“iRFP”) were treated with doxycycline for 18 h to induce expression of PA-X. Total cellular RNA was extracted and RT-qPCR was performed to measure levels of the indicated endogenous RNAs or the PA-X mRNA in panel C. Gene expression normalized by 18S rRNA levels is plotted as fold change in induced (PA-X- or RFP-expressing) vs. uninduced cells. The numbers indicate the clones of HEK293T and A549 iPA-X cells used for this panel. *,**,*** = *p* value (Student’s *t*-test) < 0.05, 0.01, 0.001 vs. 293T iRFP (A, n = 5) or A549 iPA-X D108A #8, shown in Fig 1C (B-C, n = 3). Error bars = s.e.m.
(EPS)

S2 Fig PA-X also targets Pol II-transcribed mRNAs that are not translated. (A) 293T cells were transfected with Pol II-driven GFP reporters and either PA-X or an empty vector. The hp-GFP construct contains a hairpin close to the 5’ end of the GFP mRNA that blocks its translation. The levels of expression of GFP protein were examined by western blotting. Tubulin is included as a loading control. A representative blot is shown. (B) 293T iPA-X cells (line #7) were transfected with a T7 polymerase expressing construct and an EMCV IRES-containing luciferase reporter driven by the T7 promoter, which results in T7-mediated RNA transcription. PA-X expression was induced 5 h after transfection by doxycycline addition for 18 h. Luciferase activity was measured using a luminescence assay and is plotted as fold change relative to background luminescence levels in cells transfected with the EMCV-luciferase construct alone. Error bars = std. dev. (C-D) HEK 293T (C) or A549 (D) cells expressing doxycycline-inducible wild-type PA-X-myc (“iPA-X wt”), a catalytically inactive mutant (“iPA-X D108A”) or RFP (“iRFP”) were treated with doxycycline for 18 h to induce expression of PA-X. Total cellular RNA was extracted and RT-qPCR was performed to measure levels of the indicated endogenous RNAs. Gene expression normalized by 18S rRNA levels is plotted as fold change in induced (PA-X- or RFP-expressing) vs. uninduced cells. The numbers indicate the clones of HEK293T and A549 iPA-X cells used for this panel. Error bars = s.e.m. *,**,*** = *p* value (Student’s *t*-test) < 0.05, 0.01, 0.001 vs. 293T iRFP (C, n = 5) or A549 iPA-X D108A #8, shown in Fig 3D (D, n = 3). (E) Expression of PA-X in the A549 iPA-X wt line #10 and D108A line #8 was induced by addition of doxycycline for 4.5 h, followed by addition of actinomycin D to stop transcription. RNA was harvested at 0,2,4,6 h after actinomycin D treatment and RT-qPCR was performed to measure levels of the indicated endogenous RNAs. Gene expression normalized by 18S rRNA levels is plotted as the log base 2 of the fold change from 0 h levels. The half-life was calculated as the inverse of the slope of the regression. In the presence of PA-X, the half-life of actin mRNA was 6.6 h and that of MALAT1 ncRNA 7.5 h. Half-lives for the RNAs in the absence of PA-X could not be calculated because there was no detectable decrease in the RNA levels over the 6-h time course of the experiment. Error bars = st. dev.
(EPS)

S3 Fig Fractionation reveals that PA-X targets both cytoplasmic and nuclear pools of mRNA. (A) PA-X targets both the cytoplasmic and the nuclear pools of susceptible reporter RNAs, and does not degrade the HR bearing constructs in either fraction. HEK 293Ts were transfected with indicated Pol-II driven GFP reporters and either PA-X or empty vector. Total cellular RNA was extracted from nuclear and cytoplasmic fraction and GFP mRNA, 7SL and U2 ncRNA levels were measured by northern blotting. A representative blot is shown. (B) Relative levels of pre-mRNAs and MALAT1 ncRNA in the nuclear vs. cytoplasmic fraction confirm that they are enriched in the nucleus (control for Fig 7A and 7B). A549 cells expressing doxycycline-inducible wild-type PA-X-myc (“iPA-X wt”, line #10) or a catalytically inactive mutant (“iPA-X D108A”, line #8) were treated with doxycycline for 18 h to induce expression of PA-X or left untreated. Total RNA was isolated from either nuclear or cytoplasmic fraction and RT-qPCR was performed to measure levels of the indicated endogenous RNAs on equal amounts of RNA. The ratio of nuclear/cytoplasmic RNA levels was calculated as the levels in the nucleus (prior to any normalization) divided by levels in the cytoplasm multiplied by 5 to correct for the relative difference in RNA isolated from the two fractions. Error bar = s.e.m., n = 3.
(EPS)

S4 Fig PA-X mediated shutoff of the reporter protein expression correlates with nuclear accumulation of the PA-X-GFP fusion constructs. (A and B) Spontaneously immortalized mouse embryonic fibroblast (MEF) cells were co-transfected with the NF-kB promoter-driven Firefly luciferase reporter (pGL4.32, Promega), thymidine kinase promoter-driven Renilla luciferase reporter (pGL4.74, Promega), and the indicated GFP-fusion protein expression constructs. At 24 h post-transfection, (A) the cells were fixed with 4% paraformaldehyde, stained with Hoechst dye to visualize cell nuclei, and analyzed by fluorescence microscopy. Asterisk indicates 2-times longer exposure for cells transfected with the PA-X-GFP fusion construct due to low level of expression. Scale bar = 20 um. Alternatively, (B) the cells were used to measure Firefly (FF-luc) and Renilla luciferase expression using Dual-Luciferase Reporter Assay kit (Promega). Values are normalized to vector-transfected cells. (C) HeLa Tet-Off cells (Clontech) were co-transfected with tetracycline response element (TRE2) promoter-driven Firefly (FF-luc) and Renilla (Renilla) luciferase reporters and the indicated GFP-fusion protein expression constructs. Luciferase activity was measured at 24 h post-transfection as described in (B). Values are normalized to those in cells transfected with the GFP-X41 fusion construct. In (B) and (C) the error bars represent standard deviation between measurements from 2 independently transfected wells.
(TIF)

S1 Table Table of primer sequences used for qPCR. (PDF)
