# Supplementary figures and images for: Selective Degradation of Host RNA Polymerase II Transcripts by Influenza A Virus PA-X Host Shutoff Protein
Source: PLoS Pathog. 2016 Feb 5;12(2):e1005427. doi: 10.1371/journal.ppat.1005427 (PMC4744033; doi:10.1371/journal.ppat.1005427)

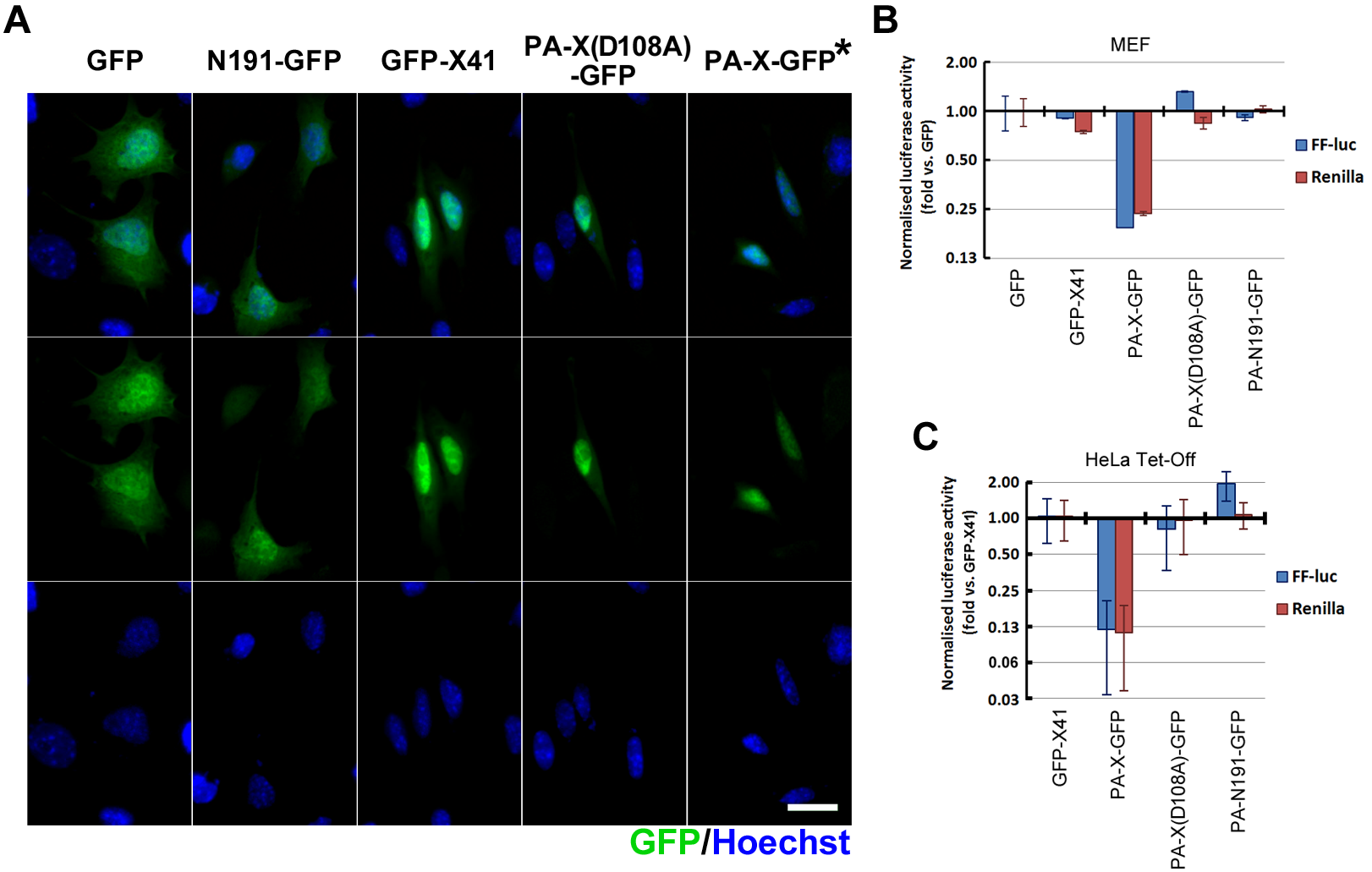

Supplement: S4 Fig — (A and B) Spontaneously immortalized mouse embryonic fibroblast (MEF) cells were co-transfected with the NF-kB promoter-driven Firefly luciferase reporter (pGL4.32, Promega), thymidine kinase promoter-driven Renilla luciferase reporter (pGL4.74, Promega), and the indicated GFP-fusion protein expression constructs. At 24 h post-transfection, (A) the cells were fixed with 4% paraformaldehyde, stained with Hoechst dye to visualize cell nuclei, and analyzed by fluorescence microscopy. Asterisk indicates 2-times longer exposure for cells transfected with the PA-X-GFP fusion construct due to low level of expression. Scale bar = 20 um. Alternatively, (B) the cells were used to measure Firefly (FF-luc) and Renilla luciferase expression using Dual-Luciferase Reporter Assay kit (Promega). Values are normalized to vector-transfected cells. (C) HeLa Tet-Off cells (Clontech) were co-transfected with tetracycline response element (TRE2) promoter-driven Firefly (FF-luc) and Renilla (Renilla) luciferase reporters and the indicated GFP-fusion protein expression constructs. Luciferase activity was measured at 24 h post-transfection as described in (B). Values are normalized to those in cells transfected with the GFP-X41 fusion construct. In (B) and (C) the error bars represent standard deviation between measurements from 2 independently transfected wells. (TIF) [file ppat.1005427.s004.tif]
