# Supplementary material for: Selective Degradation of Host RNA Polymerase II Transcripts by Influenza A Virus PA-X Host Shutoff Protein
Source: PLoS Pathog. 2016 Feb 5;12(2):e1005427. doi: 10.1371/journal.ppat.1005427 (PMC4744033; doi:10.1371/journal.ppat.1005427)
Supplement: S1 Table — (PDF) [file ppat.1005427.s005.pdf]

Table S1. Primers used for qPCR

qPCR primer	Sequence (5'-3')	source
GFP	F- CAACAGCCACAACGTCTATATCATG<br>R- ATGTTGTGGCGGATCTTGAAG	Lee and Glaunsinger, PLOS Biology, 2009
DsRed	F-GATCCACAAGGCCCTGAAGC<br>R-GCTCCACGATGGTGTAGTCC	Janus et al., Appl Env Microbiology, 2007
18S rRNA	F- GTAACCCGTTGAACCCCATT<br>R- CCATCCAATCGGTAGTAGCG	Abernathy et al., Cell Host Microbe, 2015
47S rRNA	F- TGTCAGGCGTTCTCGTCTC<br>R- AGCACGACGTCACCACATC	Feng et al., Nat Struct Mol Biol, 2010
RPS18	F- GAT GGGCGGCGGAAAATAG<br>R- GCGTGGATTCTGCATAATGGT	Mak et al., Biochemistry Research International, 2011
RPS6	F- CGATGAACGCAAACTTCGTA<br>R- TTCGGACCACATAACCCTTC	Hagner et al., Oncogene, 2011
Beta-actin	F- CGTCACCAACTGGGACGACA<br>R- CGTCACCAACTGGGACGACA	Feng et al., Nat Struct Mol Biol, 2010
GAPDH	F- AGCCACATCGCTCAGACAC<br>R- TGGAAGATGGTGATGGGATT	Arias et al., PLOS Pathogens, 2014
POLR2A	F- GAGAGTCCAGTTCGGAGTCCT<br>R- CCCTCAGTCGTCTCTGGGTA	Farrall et al., Oncogene, 2009
7SK	F- CTCCAAACAAGCTCTCAAGGTCCA<br>R- ATGCAGCGCCTCATTTGGATGTGT	Barboric et al., PNAS, 2009
7SL	F- GGAGTTCTGGGCTGTAGTGC<br>R- GGAGTTCTGGGCTGTAGTGC	Ren et al., PLOS ONE, 2012
5S	F-GGCCATACCACCCTGAACGC<br>R-CAGCACCCGGTATTCCCAGG	Thomas et al., Cell Reports, 2015
U2	F- TGGAGCAGGGAGATGGAATA<br>R- CGTTCCTGGAGGTACTGCAA	Thomas et al., Cell Reports, 2015
MALAT1	F-GACGGAGGTTGAGATGAAGC<br>R-ATTCGGGGCTCTGTAGTCCT	Tripathi et al., PLOS Genetics, 2013
GusB	F- CTCATTTGGAATTTTGCCGATT<br>R- CCGAGTGAAGATCCCCTTTTTA	Abernathy et al., Cell Host Microbe, 2015
EEF1A	F- TGTCGTCATTGGACACGTAGA<br>R- ACGCTCAGCTTTCAGTTTATCC	Abernathy et al., Cell Host Microbe, 2015
Beta-tubulin	F- TCTACCTCCCTCACTCAGCT<br>R- CCAGAGTCAGGGGTGTTCAT	This paper
GAPDH – intronic	F- TCCCCTCCTCATGCCTTCTT<br>R- CCAGGCGCCCAATACG	Sei et al., PLOS Pathogens, 2015
Beta-actin - intronic	F- AGGGCTTCTTGTCCTTTCCT<br>R- CATAGGAATCCTTCTGACCCA	Sei et al., PLOS Pathogens, 2015

HIST1H3C	F- GCTTGCTACTAAAGCAGCCC<br>R- AGCGCACAGATTGGTGTCTTC	Harvard primer bank ID 21071022c1; Spandidos et al., 2010
PR8 PA-X	F-GCGACAATGCTTCAATCCGA<br>R-TTGACTCGCCTTGCTCATTG	This paper
Luciferase	F- ATGGAAGACGCCAAAAACAT<br>R- GCCTTATGCAGTTGCTCTCC	This paper
TP53TG1	F-ACGAAGGTACCCAACCCTCT<br>R-GGTGTAAGTGTTCGCCTGGT	Yang et al., PLOS ONE, 2013
PR8 PA	F-TCTCAGCGGTCCAAATTCCT<br>R-TCTGCCAGTACTTGCTTCCA	This paper
PR8 NP	F- CCCAGGATGTGCTCTCTGAT<br>R- TTCGTCCATTCTCACCCCTC	This paper
PR8 M1	F- TTTGGCCTGGTATGTGCAAC<br>R- ACCATTTGCCTAGCCTGACT	This paper
PR8 M2	F- GGTCGAAACGCCTATCAGAA<br>R- ACTTTGGCACTCCTTCCGTA	This paper
PR8 NS1	F- CTGTGTCAAGCTTTCAGGTAGA<br>R- GGTACAGAGGCCATGGTCAT	This paper
PR8 NEP	F- CTGTGTCAAGCTTTCAGGACA<br>R- TTGTTCCCGCCATTTCTC	This paper
